# Early ACEI/ARB use and in-hospital outcomes of acute myocardial infarction patients with systolic blood pressure <100 mmHg and undergoing percutaneous coronary intervention: Findings from the CCC-ACS project

**DOI:** 10.3389/fcvm.2022.1003442

**Published:** 2022-09-29

**Authors:** Xuedong Zhao, Guanqi Zhao, Mengge Zhou, Ge Wang, Changsheng Ma, Sidney C. Smith, Gregg C. Fonarow, Louise Morgan, Bin Que, Hui Ai, Jing Liu, Dong Zhao, Shaoping Nie

**Affiliations:** ^1^Division of Cardiology, Center for Coronary Artery Disease, Beijing Anzhen Hospital, Capital Medical University, Beijing, China; ^2^Vanke School of Public Health, Tsinghua University, Beijing, China; ^3^Division of Cardiology, Arrhythmia Center, Beijing Anzhen Hospital, Capital Medical University, Beijing, China; ^4^Division of Cardiology, University of North Carolina at Chapel Hill, Chapel Hill, NC, United States; ^5^Division of Cardiology, Geffen School of Medicine at University of California, Los Angeles, Los Angeles, CA, United States; ^6^Department of International Quality Improvement, American Heart Association, Dallas, TX, United States; ^7^The Key Laboratory of Remodeling-Related Cardiovascular Diseases, Ministry of Education, Department of Epidemiology, Beijing Anzhen Hospital, Beijing Institute of Heart, Lung and Blood Vessel Diseases, Capital Medical University, Beijing, China

**Keywords:** ACEI/ARB, AMI, low blood pressure, PCI, mortality, CCC-ACS

## Abstract

**Background:**

Few studies have evaluated whether acute myocardial infarction (AMI) patients with relatively low blood pressure benefit from early ACEI/ARB use in the era of percutaneous coronary intervention (PCI).

**Objectives:**

This study evaluated the associations of ACEI/ARB use within 24 h of admission with in-hospital outcomes among AMI patients with SBP < 100 mmHg and undergoing PCI.

**Methods:**

This study was based on the Improving Care for Cardiovascular Disease in China-ACS project, a collaborative registry and quality improvement project of the American Heart Association and the Chinese Society of Cardiology. Between November 2014 and December 2019, a total of 94,623 patients with AMI were enrolled. Of them, 4,478 AMI patients with SBP < 100 mmHg and undergoing PCI but without clinically diagnosed cardiogenic shock at admission were included. Multivariable logistic regression and propensity score-matching analysis were used to evaluate the association between early ACEI/ARB use and in-hospital major adverse cardiac events (MACEs), a combination of all-cause death, cardiogenic shock, and cardiac arrest.

**Results:**

Of AMI patients, 24.41% (*n* = 1,093) were prescribed ACEIs/ARBs within 24 h of admission. Patients with early ACEI/ARB use had a significantly lower rate of MACEs than those without ACEI/ARB use (1.67% vs. 3.66%, *p* = 0.001). In the logistic regression analysis, early ACEI/ARB use was associated with a 45% lower risk of MACEs (odds ratio: 0.55, 95% CI: 0.33–0.93; *p* = 0.027). Further propensity score-matching analysis still showed that patients with early ACEI/ARB use had a lower rate of MACEs (1.96% vs. 3.93%, *p* = 0.009).

**Conclusion:**

This study found that among AMI patients with an admission SBP < 100 mmHg undergoing PCI, early ACEI/ARB use was associated with better in-hospital outcomes. Additional studies of the early use of ACEIs/ARBs in AMI patients with relatively low blood pressure are warranted.

## Graphical Abstract

Acute myocardial infarction patients with relatively low blood pressure derive benefits from early ACEI/ARB use in the era of percutaneous coronary intervention. 
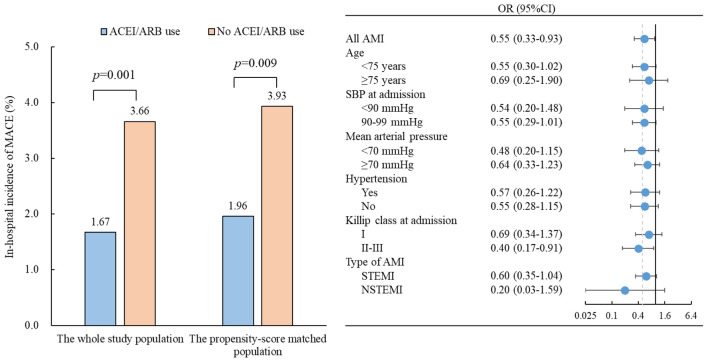

## Introduction

Initiating angiotensin-converting enzyme inhibitors (ACEIs) or angiotensin receptor blockers (ARBs) early after acute myocardial infarction (AMI) has been well proven to improve the prognosis of those patients in landmark clinical trials (1–6). However, most of these trials excluded patients with systolic blood pressure (SBP) < 100 mmHg for concerns about hypotension and hemodynamic instability (1–4). Guidelines have recommended that ACEI/ARB use should be avoided or used with caution in the first 24 h of AMI in patients with hypotension (7–10). To date, few studies have evaluated whether AMI patients with SBP < 100 mmHg but without severe contraindications at admission, such as cardiogenic shock, could derive clinical benefits from early ACEI/ARB use. In addition, most studies that evaluated the effect of ACEIs/ARBs were conducted in the 1990s, when percutaneous coronary intervention (PCI) was not widely applied in clinical practice (1–4, 11, 12). Therefore, this study aimed to evaluate the association between the use of ACEIs/ARBs within 24 h of admission and in-hospital outcomes among AMI patients with SBP < 100 mmHg and undergoing PCI.

## Materials and methods

### Study design

The Improving Care for Cardiovascular Disease in China–Acute Coronary Syndrome (CCC-ACS) project is a collaborative initiative of the American Heart Association and the Chinese Society of Cardiology. It is a large nationwide registry and quality-improvement study launched in 2014 in China, focusing on improving the medical quality of ACS care. The details of the design and methodology of the CCC-ACS project were published in 2016 (13, 14). Briefly, a total of 241 hospitals nationwide were included in this study, including 159 tertiary hospitals and 82 secondary hospitals. According to the research manual, all required data based on medical records should be reported *via* a web-based data-collection platform (Oracle Clinical Remote Data Capture; Oracle Corp., Redwood City, CA, USA) by trained data abstractors. Third-party clinical research associates were hired to audit the inclusion of cases, ensuring that the cases were reported consecutively rather than selectively. Approximately 5% of the cases were randomly selected for comparison with the original records to assess the accuracy and completeness of the reported data.

### Study population

Between November 2014 and December 2019, a total of 94,623 patients with a definite principal diagnosis of ST-elevation myocardial infarction (STEMI) and non-ST-elevation myocardial infarction (NSTEMI) were enrolled in this study. STEMI and NSTEMI were defined according to respective guidelines issued by the Chinese Society of Cardiology (15, 16). The diagnostic criteria for AMI were based on chest pain or discomfort, ECG, and measurements of myocardial injury biomarkers (14). Among these AMI patients, 7,763 had SBP less than 100 mmHg at admission. After excluding patients with clinically diagnosed cardiogenic shock at admission (*n* = 1,884) and those who did not undergo PCI during hospitalization (*n* = 1,401), 4,478 patients with a definite diagnosis of AMI were included in this study (Supplementary Figure 1).

### Study variables

#### ACEI/ARB use

Information about ACEI/ARB use was obtained based on patients’ medical records, including whether patients used ACEIs/ARBs within 24 h of hospitalization and whether ACEIs/ARBs were prescribed at discharge. Taking ACEIs/ARBs within 24 h of hospitalization was defined as early ACEI/ARB use.

#### In-hospital outcomes

The outcome of this study was a composite of major adverse cardiac events (MACEs) that occurred during hospitalization, including all-cause death, cardiogenic shock, and cardiac arrest. Items for diagnosing cardiogenic shock in clinical practice generally included lower SBP (< 90 mmHg) with appropriate fluid resuscitation with clinical and laboratory evidence of end-organ damage. The clinical manifestations included altered mental status, cold extremities, oliguria, and narrow pulse pressure. Laboratory indicators included metabolic acidosis, elevated serum lactate, elevated serum creatinine, etc. Patients with sudden loss of consciousness accompanied by convulsions, loss of heart sounds, undetectable pulse and blood pressure and other symptoms can be diagnosed with cardiac arrest.

#### Definition of other variables

Hypertension was defined as having self-reported physician-diagnosed hypertension or receiving antihypertensive therapy before hospitalization. Admission mean arterial pressure (MAP) was calculated by admission SBP and diastolic blood pressure (DBP), i.e., MAP = 1/3*SBP + 2/3*DBP. Diabetes mellitus was defined as having a self-reported physician-diagnosed diabetes mellitus, receiving oral hypoglycemic drug therapy or insulin therapy, or having a fasting blood glucose level ≥ 7.0 mmol/L (126 mg/dL) or hemoglobin A1c concentration ≥ 6.5%. Elevated low-density lipoprotein cholesterol (LDL-C) was defined as a serum LDL-C level ≥ 1.8 mmol/L (70 mg/dL) (17). Low high-density lipoprotein cholesterol (HDL-C) was defined as serum HDL-C < 1.0 mmol/L (40 mg/dL) (17). Elevated triglyceride (TG) was defined as serum TG ≥ 2.3 mmol/L (200 mg/dL) (17). The estimated glomerular filtration rate (eGFR) was calculated by the equation developed by the Chronic Kidney Disease Epidemiology Collaboration (18) and then divided into 4 groups: below 30, 30–59, 60–89 and ≥ 90 ml/min/1.73 m^2^. A history of coronary heart disease (CHD) was defined if the patient had a history of myocardial infarction or underwent PCI or coronary artery bypass grafting before hospitalization. Other medical histories, including heart failure, atrial fibrillation, cerebrovascular disease, and renal failure, were defined according to the original notes of medical records. Severe manifestations at admission, including acute heart failure and cardiac arrest, were defined based on the documentation of the clinical condition at admission in medical records (14). In-hospital treatments were also judged according to the original medical records. In-hospital dual antiplatelet therapy (DAPT) was defined if patients used both aspirin and P2Y_12_ inhibitors.

### Statistical analysis

Continuous variables are shown as the mean (standard deviation [SD]) or median (interquartile range), and differences between groups were compared using *t-test*s or the *Mann–Whitney U*-test according to the distribution; categorical variables are presented as the number (percentage) and were compared using the *chi*-squared test. Logistic regression analysis was carried out to evaluate the effect of early ACEI/ARB use on in-hospital MACEs. The adjusted variables included patient age and sex, admission SBP, heart rate, renal insufficiency, first Killip class, history of hypertension, diabetes, coronary heart disease, cerebrovascular disease, in-hospital treatment with DAPT, statins, β-blockers, and type of AMI. For analysis of the association between ACEIs/ARBs and MACEs, patients with cardiac arrest at admission were excluded. Odds ratios (ORs) with 95% confidence intervals (CIs) were reported.

In addition, we analyzed the association between ACEIs and ARBs and in-hospital MACEs separately. Subgroup analyses of age (<75/≥75 years), admission SBP (<90/90–99 mmHg), MAP (<70/≥70 mmHg), history of hypertension (yes/no), Killip class at admission (I/II-III), and type of AMI (STEMI/NSTEMI) were also performed, with multivariable adjustment in the logistic regression model. In addition, we excluded patients who died within 24 h of hospitalization for sensitivity analysis.

A propensity score-matched analysis was further conducted to re-evaluate the effect of early ACEI/ARB use on in-hospital outcomes. First, a propensity score of early ACEI/ARB use was calculated by a logistic regression model with variables of the number of hospitals, level of hospitals, patients’ age, sex, levels of SBP, heart rate, eGFR, first Killip class, history of hypertension, diabetes mellitus, coronary heart disease, cerebrovascular disease, heart failure, renal failure, prehospital treatment of aspirin, P2Y_12_ inhibitors, β-blockers and ACEI/ARB, in-hospital DAPT, statins, β-blockers and type of ACS. Patients with and without early ACEI/ARB use were then matched at a 1:1 ratio by propensity score using nearest-neighbor matching without replacement with a caliper of 0.02. The absolute standardized differences of variables included for the calculation of propensity score were calculated before and after propensity score matching. The absolute standardized differences < 10.0% for variables indicated a relatively small imbalance. In-hospital outcomes were presented as the number (percentage) and compared using a *chi*-squared test for paired data. Univariate logistic regression was conducted to calculate the OR and 95% CI.

Variables with missing data were imputed by the sequential regression multiple imputation method using IVEware software version 0.2 (Survey Research Center, University of Michigan, MI, USA) in the total ACS population of the CCC-ACS project.

Statistical analyses were performed using SAS 9.4 (SAS Institute, Cary, NC, USA) and Stata 14.0 (Stata, College Station, TX, USA). Two-tailed *P*-values of < 0.05 were considered statistically significant.

## Results

### Comparison of characteristics and treatment between patients with and without early ACEI/ARB use

Among the 4,478 AMI patients included in this study, only 1,093 (24.41%) were prescribed ACEIs/ARBs at admission, with 424 (9.47%) being ACEIs and 669 (14.94%) being ARBs. The characteristics were generally similar, except for the prevalence of hypertension, between patients with and without ACEI/ARB use (Table 1). In addition, patients with ACEI/ARB use had a more active in-hospital treatment of β-blockers (67.7% vs. 31.5%, *P* < 0.001) (Table 1).

**TABLE 1:** Comparison of characteristics and in-hospital treatment between patients with and without early ACEI/ARB use.

	ACEI/ARB use (*n* = 1,093)	No ACEI/ARB use (*n* = 3,385)	*P*-value
Age, mean (*SD*), years	61.12 (11.33)	62.12 (11.91)	0.012
Female, *n* (%)	204 (18.66)	637 (18.82)	0.910
Vital signs			
SBP levels, mean (*SD*), mmHg	92.5 (6.10)	91.77 (6.65)	0.001
DBP levels, mean (*SD*), mmHg	60.14 (7.12)	59.21 (7.57)	<0.001
MAP levels, mean (*SD*), mmHg	70.93 (5.96)	70.06 (6.45)	<0.001
Heart rates, mean (*SD*), bpm	74.85 (16.06)	74.15 (18.47)	0.225
Risk factors			
Hypertension, *n* (%)	464 (42.45)	1,089 (32.17)	<0.001
Diabetes mellitus, *n* (%)	455 (41.63)	1,398 (41.3)	0.848
Elevated LDL-C, *n* (%)	922 (84.35)	2,842 (83.96)	0.758
Low HDL-C, *n* (%)	551 (50.41)	1,488 (43.96)	<0.001
Elevated TG, *n* (%)	203 (18.57)	556 (16.43)	0.100
eGFR, *n* (%)			0.389
< 30 ml/min/1.73 m^2^	23 (2.1)	88 (2.6)	
30–59 ml/min/1.73 m^2^	144 (13.17)	501 (14.8)	
60–89 ml/min/1.73 m^2^	399 (36.51)	1,230 (36.34)	
≥90 ml/min/1.73 m^2^	527 (48.22)	1,566 (46.26)	
History of diseases			
CHD, *n* (%)	61 (5.58)	187 (5.52)	0.943
Heart failure, *n* (%)	8 (0.53)	18 (0.73)	0.449
Atrial fibrillation, *n* (%)	10 (0.91)	49 (1.45)	0.179
Cerebrovascular disease, *n* (%)	52 (4.76)	209 (6.17)	0.082
Renal failure, *n* (%)	9 (0.82)	31 (0.92)	0.080
Critical cardiac symptoms at admission			
Heart failure, *n* (%)	52 (4.76)	145 (4.28)	0.507
Cardiac arrest, *n* (%)	15 (1.37)	53 (1.57)	0.207
Prehospital treatment			
Aspirin, *n* (%)	200 (18.3)	544 (16.07)	0.085
P2Y_12_ inhibitors, *n* (%)	171 (15.65)	422 (12.47)	0.007
Statins, *n* (%)	131 (11.99)	400 (11.82)	0.881
Beta-blockers, *n* (%)	72 (6.59)	123 (3.63)	<0.001
ACEI/ARB, *n* (%)	126 (11.53)	83 (2.45)	<0.001
In-hospital treatment			
DAPT, *n* (%)	1,063 (97.26)	3,247 (95.92)	0.044
Statins, *n* (%)	1,068 (97.71)	3,180 (93.94)	<0.001
Beta-blockers, *n* (%)	740 (67.7)	1,065 (31.46)	<0.001
Type of AMI, *n* (%)			0.356
STEMI	914 (83.62)	2,870 (84.79)	
NSTEMI	179 (16.38)	515 (15.21)	

ACEI, angiotensin-converting enzyme inhibitor; ACS, acute coronary syndrome; ARB, angiotensin receptor blocker; DAPT, dual antiplatelet therapy; NSTEMI, non-ST-elevation myocardial infarction; PCI, percutaneous coronary intervention; SBP, systolic blood pressure; *SD*, standard deviation; STEMI, T-elevation myocardial infarction.

### Association between early ACEI/ARB use and in-hospital outcomes

**FIGURE 1:**
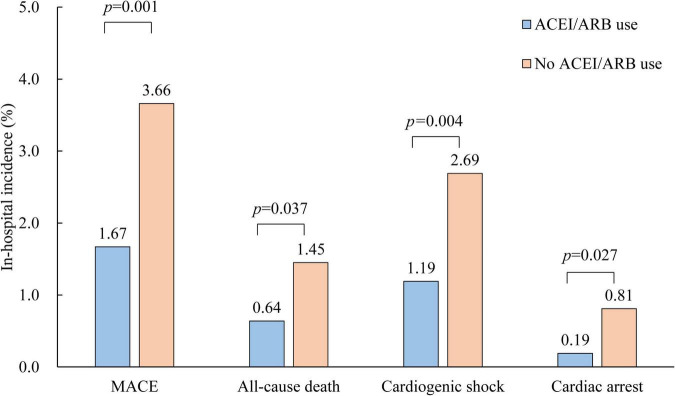
Comparison of in-hospital outcomes of AMI patients with and without early ACEI/ARB use. ACEI, angiotensin-converting enzyme inhibitor; ARB, angiotensin receptor blocker.

**FIGURE 2:**
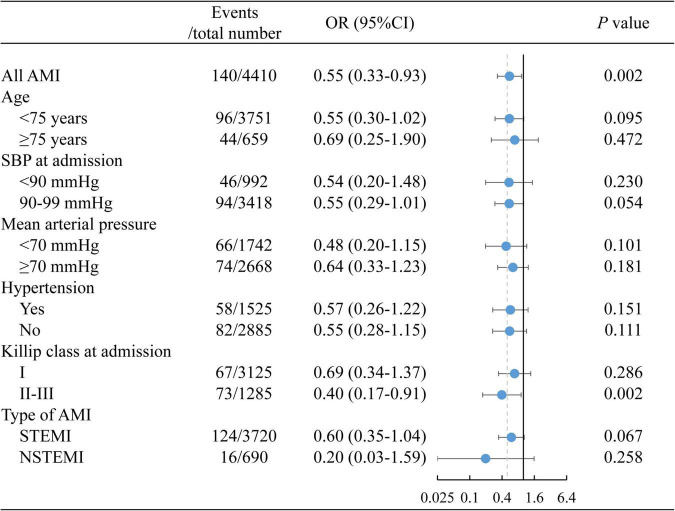
The association between early ACEI/ARB use and in-hospital MACEs. ACEI, angiotensin-converting enzyme inhibitor; ARB, angiotensin receptor blocker; MACEs, major adverse cardiac events.

Patients with early ACEI/ARB use had lower incidences of in-hospital MACE (1.67% vs. 3.66%, *p* = 0.001), all-cause death (0.64% vs. 1.45%, *p* = 0.037), cardiogenic shock (1.19% vs. 2.69%, *p* = 0.004) and cardiac arrest (0.19% vs. 0.81%, *p* = 0.027) (Figure 1). Multivariate-adjusted analysis was then conducted to evaluate the independent association between early ACEI/ARB use and in-hospital MACEs. Early ACEI/ARB use was significantly associated with a lower risk of MACEs (OR: 0.55, 95% CI: 0.33–0.93; *p* = 0.027) (Figure 2). Subgroup analysis of age, admission SBP, admission MVP, hypertension, Killip class at admission, and types of AMI consistently showed that early ACEI/ARB use was associated with a reduced risk of MACEs (Figure 2), although without statistical significance among some subgroups.

Patients who died within 24 h were then excluded from the sensitivity analysis, which also showed that patients with early ACEI/ARB use had a lower risk of MACEs (OR: 0.57; 95% CI: 0.34–0.98; *p* = 0.040).

We further separately analyzed the association between ACEIs and ARBs and in-hospital MACEs. The association between early ACEI use and MACEs was not statistically significant (OR: 0.65, 95% CI: 0.32–1.32; *p* = 0.229); however, the early use of ARBs was still significantly associated with a reduced risk of MACEs (OR: 0.48, 95% CI: 0.24–0.97; *p* = 0.042).

**FIGURE 3:**
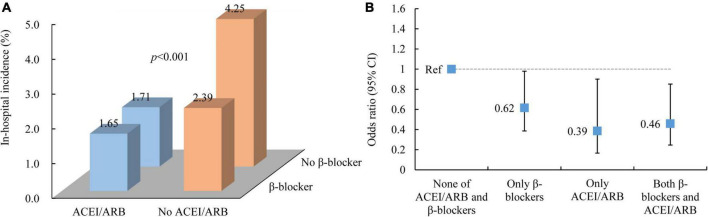
In-hospital outcomes of AMI patients by early ACEI/ARB and β-blocker use. **(A)** In-hospital incidence of MACEs. **(B)** The association between ACEI/ARB and β-blocker use and in-hospital MACEs. ACEI, angiotensin-converting enzyme inhibitor; ARB, angiotensin receptor blocker; MACEs, major adverse cardiac events.

As we found that patients using ACEIs/ARBs were also more inclined to use β-blockers at the same time, we further divided the use of ACEIs/ARBs and β-blockers into four groups for *post hoc* analysis: no ACEIs/ARBs or β-blockers, only ACEIs/ARBs, only β-blockers and both ACEIs/ARBs and β-blockers. This analysis showed that patients with no ACEIs/ARBs or β-blockers had the highest incidence of in-hospital MACEs (4.25%), followed by patients only using β-blockers (2.39%). Patients using ACEIs/ARBs with and without β-blocker cotreatment had a similar incidence of MACEs (with β-blockers: 1.65%; without β-blockers: 1.71%) (Figure 3A). Compared with patients with no ACEIs/ARBs or β-blockers, patients using only ACEIs/ARBs (OR: 0.39, 95% CI: 0.17–0.90; *p* = 0.028), those using only β-blockers (OR: 0.62, 95% CI: 0.39–0.98; *p* = 0.041), and those using both ACEIs/ARBs and β-blockers (OR: 0.46, 95% CI: 0.25–0.85; *p* = 0.014) all exhibited a significantly reduced risk of MACEs (Figure 3B).

### Propensity score-matching analysis

After propensity score matching, 1,019 patients with early ACEI/ARB use were matched with 1,019 patients without ACEI/ARB use. The absolute standardized differences of all variables included for the calculation of propensity score were less than 10.0%, indicating that AMI patients with and without early ACEI/ARB use were well matched (Supplementary Figure 2). The rates of MACEs remained lower in patients with early ACEI/ARB use after matching (1.96% vs. 3.93%, *p* = 0.009) than in those without ACEI/ARB use. Patients with early ACEI/ARB use still had a lower risk of MACEs (OR: 0.49, 95% CI: 0.28–0.84; *p* = 0.010).

### Discharge ACEI/ARB use among surviving acute myocardial infarction patients

**FIGURE 4:**
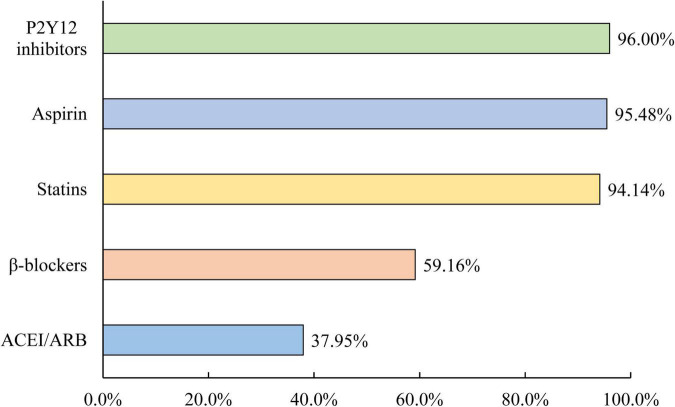
Discharge mediations for the secondary prevention of MI.

Discharge prescriptions of ACEIs/ARBs were evaluated in 4,422 survivors of AMI. Of these patients, only 37.95% received an ACEI/ARB prescription at discharge, which was still much lower than other recommended drugs for secondary prevention, including 95.48% for aspirin, 96.00% for P2Y_12_ inhibitors, 94.14% for statins, and 59.16% for β-blockers (Figure 4). Only 20.23% were prescribed ACEIs/ARBs both within 24 h of admission and at discharge, and 58.31% of them were not prescribed ACEIs/ARBs within 24 h of admission or at discharge.

## Discussion

To our knowledge, this is the first study that specifically evaluated the associations of early ACEI/ARB use with in-hospital MACEs among hospitalized AMI patients with an admission SBP < 100 mmHg who were undergoing PCI. We found that early ACEI/ARB use was significantly associated with a reduced risk of MACEs among those patients.

### Low rate of ACEI/ARB usage

Less than 1/4 of AMI patients with admission SBP < 100 mmHg used ACEIs/ARBs within 24 h of hospitalization, which may have resulted from the clinician’s consideration of hypotension. However, only approximately 1/3 of surviving AMI patients received prescriptions for ACEIs/ARBs at discharge, much lower than other evidence-based therapies for secondary prevention of AMI.

The China Patient-centered Evaluative Assessment of Cardiac Events Retrospective Study of AMI (China PEACE-Retrospective AMI Study) found that AMI patients with SBP < 90 mmHg were less likely to receive ACEI/ARB therapy (OR: 0.55, 95% CI: 0.42–0.71) than those with SBP between 90 and 139 mmHg (19). However, for patients with SBP between 90 and 99 mmHg, only one in four patients was prescribed ACEIs/ARBs within 24 h of admission in this study. Notably, patients with lower admission blood pressure also had lower ACEI/ARB prescription rates at discharge. Therefore, the reason for the consistently low use rate of ACEIs/ARBs among surviving patients at discharge deserves further investigation in medical improvement research.

### ACEI/ARB and improved in-hospital outcomes

In this study, we observed that early use of ACEIs/ARBs was significantly associated with a lower risk of MACEs among patients with SBP < 100 mmHg undergoing PCI, especially for patients with SBP between 90 and 99 mmHg and those who had heart failure (Killip II-III).

In 1998, a systematic review of individual data conducted by the ACE Inhibitor Myocardial Infarction Collaborative Group, based on 98,496 patients from 4 eligible trials, found that early use of ACEIs could significantly reduce the risk of mortality risk in AMI patients (5), which reinforces the important role of ACEIs in the treatment of patients with AMI and is still widely cited by guidelines or consensus to date (8, 9, 20, 21). However, the majority of AMI patients with SBP < 100 mmHg were excluded from enrollment in these trials (1–4). Eventually, 2,463 AMI patients were included in this review, accounting for only 3% of the total study population (5). In the subgroup analysis of those with SBP < 100 mmHg, it was found that patients with ACEI use had a higher incidence of 30-day mortality (13.1% vs. 12.0%), but without statistical significance. However, after more than 20 years of medical development, the mortality rate of AMI has decreased significantly (5, 22). The main intervention of reperfusion has also changed from fibrinolytic therapy to PCI (22). Therefore, there is a large clinical heterogeneity between these studies and our study. The benefit of ACEIs/ARBs observed in this study among AMI patients with SBP < 100 mmHg provides new evidence for the application of ACEIs/ARBs in clinical practice. In this study, both multivariate adjusted analysis and propensity score matching analysis consistently showed that AMI patients with early ACEI/ARB use had a lower risk of MACEs, suggesting that even patients with relatively low SBP at admission could also benefit from the early application of ACEIs/ARBs. However, in clinical practice, clinicians should comprehensively understand the causes and progression of low blood pressure in patients and should be cautious in prescribing ACEIs/ARBs if the patient has clinical manifestations of hypotension or continuously lowering blood pressure.

### ACEI vs. ARB

Although guidelines for the treatment of AMI recommended ARBs as an alternative for those who are ACEI intolerant (7–10), ARBs were more widely used than ACEIs in this study. When separately evaluating the effect of ACEIs and ARBs on in-hospital MACEs, only ARB use was significantly associated with a reduced risk of MACEs.

Previous studies have extensively compared the efficacy and safety of ACEIs and ARBs among different populations (23–27). ARBs are as effective as ACEIs but have fewer side effects. To date, ESC/ESH Guidelines for the management of hypertensive patients have considered ACEIs and ARBs to be equivalent (28). The 2021 ACC/AHA heart failure guideline updated ACEIs or ARBs as equal first-line treatments for newly diagnosed stage C heart failure with reduced ejection fraction (29). Future guidelines for AMI could consider raising the ARB recommendation level based on growing research evidence (26, 30).

### ACEIs/ARBs and β-blockers

For patients with lower blood pressure, whether to start ACEIs/ARBs earlier, β-blockers or both, is controversial in clinical practice because both drugs have antihypertensive effects. In this study, we found that there was a lower risk of either using ACEIs/ARBs, β-blockers or both. However, although the combined use of ACEIs/ARBs had the lowest incidence of MACEs in univariate analysis, it was found that the use of ACEIs/ARBs alone had the lowest odds ratio after multivariate adjustment, indicating that the combined use of ACEIs/ARBs and β-blockers did not further reduce the risk of patients. In clinical practice, physicians could prefer beta-blockers, which was also observed in this study (a higher utilization rate of β-blockers compared with ACEI/ARB), considering its cardioprotective effects on attenuating the increased sympathetic drive and thereby reducing myocardial oxygen consumption, suppressing ventricular arrhythmias (31), but a soft antihypertensive effect. However, the contemporary evidence of using β-blockers is disputable (31). A meta-analysis showed that β-blockers could reduce recurrent myocardial infarction and angina (short-term) at the expense of an increase in heart failure and cardiogenic shock but ultimately have no mortality benefit in the treatment of AMI (32). Therefore, the benefits of β-blockers for patients with relatively low blood pressure at admission undergoing PCI remain to be explored by future specially designed studies.

### Cardioprotective mechanism of ACEIs/ARBs

The benefit occurs during hospitalization for AMI with SBP < 100 mmHg, suggesting that protective mechanisms of ACEIs/ARBs other than antihypertensive effects and remodeling processes may play a role. The potential mechanisms could include an early effect on a reduction of neurohormonal activation and infarct size and an increase in regional wall motion and collateral coronary flow (33–36).

Experimental studies have shown that the expression levels of angiotensin-converting enzyme, angiotensin (Ang) II, Ang II type 1 receptor, and Ang II type 2 receptor were significantly increased within a few hours in the myocardial ischemic area with reperfusion, indicating that activation of the cardiac local renin-angiotensin system may be important in the regulation of myocardial ischemia/reperfusion injury (35, 37). Meanwhile, studies found that administration of ACEIs/ARBs could rapidly and significantly decrease the infarct size and inflammatory response and bring early benefits (35, 38–40).

### Concerns for hypotension

For AMI patients with SBP < 100 mmHg, clinicians could be concerned about the development of hypotension after using ACEIs/ARBs. However, hypotension is not the same as shock. In our study, we even found that patients with early ACEI/ARB use had a lower incidence of cardiogenic shock. The Chinese expert consensus on the application of ACEIs in patients with coronary heart disease recommended that when hypotension (SBP < 90 mmHg) occurs during ACEI therapy, ACEIs should be continued if the patient is asymptomatic (41). For patients with hypotensive symptoms, other antihypertensive drugs should first be suspended, such as nitrates and calcium antagonists (41). Therefore, the use of ACEIs/ARBs in clinical practice is necessary and should be emphasized considering their multiple cardiovascular benefits.

### Limitations

There are several limitations of this study. First, this was an observational study instead of an RCT, and uncollected confounding factors could still exist even after adjustment for multiple variables. However, it is not feasible to conduct RCTs in such a high-risk population. Evidence from real-world research is increasingly valued. Second, the limited sample size of this study did not have sufficient power to confirm the results of some important subgroup analyses. However, to our knowledge, this was the only study to date that specifically explored the role of ACEIs/ARBs in hospitalized AMI patients with SBP < 100 mmHg undergoing PCI. We expected to promote more research to focus on this special population through our study. Third, for patients with lower blood pressure, the dose of ACEIs/ARBs is very important in the early stage. The initial dose was not collected in this study, so the effect of dose could not be assessed. However, for experienced clinicians, ACEI/ARB is generally applied from a small dose according to the patient’s SBP level. Fourth, this study only evaluated the outcomes of ACEIs/ARBs during hospitalization and could not assess their long-term effects. However, treatment in the acute phase is particularly critical for those admitted with hypotension. Meanwhile, the study found an underuse of ACEIs/ARBs based on discharge prescriptions, indicating that in addition to the problem of hypotension, there were other factors that affect the use of ACEIs/ARBs, and the potential influencing factors are worthy of further study.

## Conclusion

To our knowledge, this is the first study with a relatively large sample size that specifically evaluated the effect of ACEIs/ARBs on AMI patients with relatively low blood pressure. It found that among AMI patients with admission SBP < 100 mmHg undergoing PCI, the early use of ACEIs/ARBs was associated with better in-hospital outcomes, which provided real-world evidence for the clinical application of ACEIs/ARBs and may further promote the early application of ACEIs/ARBs and eventually improve the prognosis of AMI patients, especially for those with SBP between 90 and 99 mmHg. Meanwhile, given that this observational study has some limitations, more studies are expected to support this finding.

## Data availability statement

The data analyzed in this study was subject to the following licenses/restrictions: The datasets analyzed during the current study are not publicly available because of intellectual property rights, but are available from the corresponding author on reasonable request. Requests to access these datasets should be directed to corresponding author.

## Ethics statement

The studies involving human participants were reviewed and approved by The Ethics Committee of Beijing Anzhen Hospital, Capital Medical University. Written informed consent for participation was not required for this study in accordance with the national legislation and the institutional requirements.

## Author contributions

XZ, GZ, and SN contributed to the conception and design of the study. GZ analyzed the data. XZ and MZ wrote the initial draft. All authors reviewed, edited, and revised the manuscript and approved it for publication.
