# Supplementary material for: Early ACEI/ARB use and in-hospital outcomes of acute myocardial infarction patients with systolic blood pressure <100 mmHg and undergoing percutaneous coronary intervention: Findings from the CCC-ACS project
Source: Front Cardiovasc Med. 2022 Sep 29;9:1003442. doi: 10.3389/fcvm.2022.1003442 (PMC9558728; doi:10.3389/fcvm.2022.1003442)
Supplement: Supplementary file 1 [file Presentation_1.pdf]

Early ACEI/ARB use and in-hospital outcomes of AMI patients with SBP <100 mmHg and undergoing PCI: findings from the CCC-ACS project

Running title: Early ACEI/ARB use in AMI patients with SBP <100 mmHg

Contents

Supplementary Figure 1 Flow chart of study population 2

Supplementary Figure 2 Absolute Standardized Differences before and after Propensity Score Matching 3

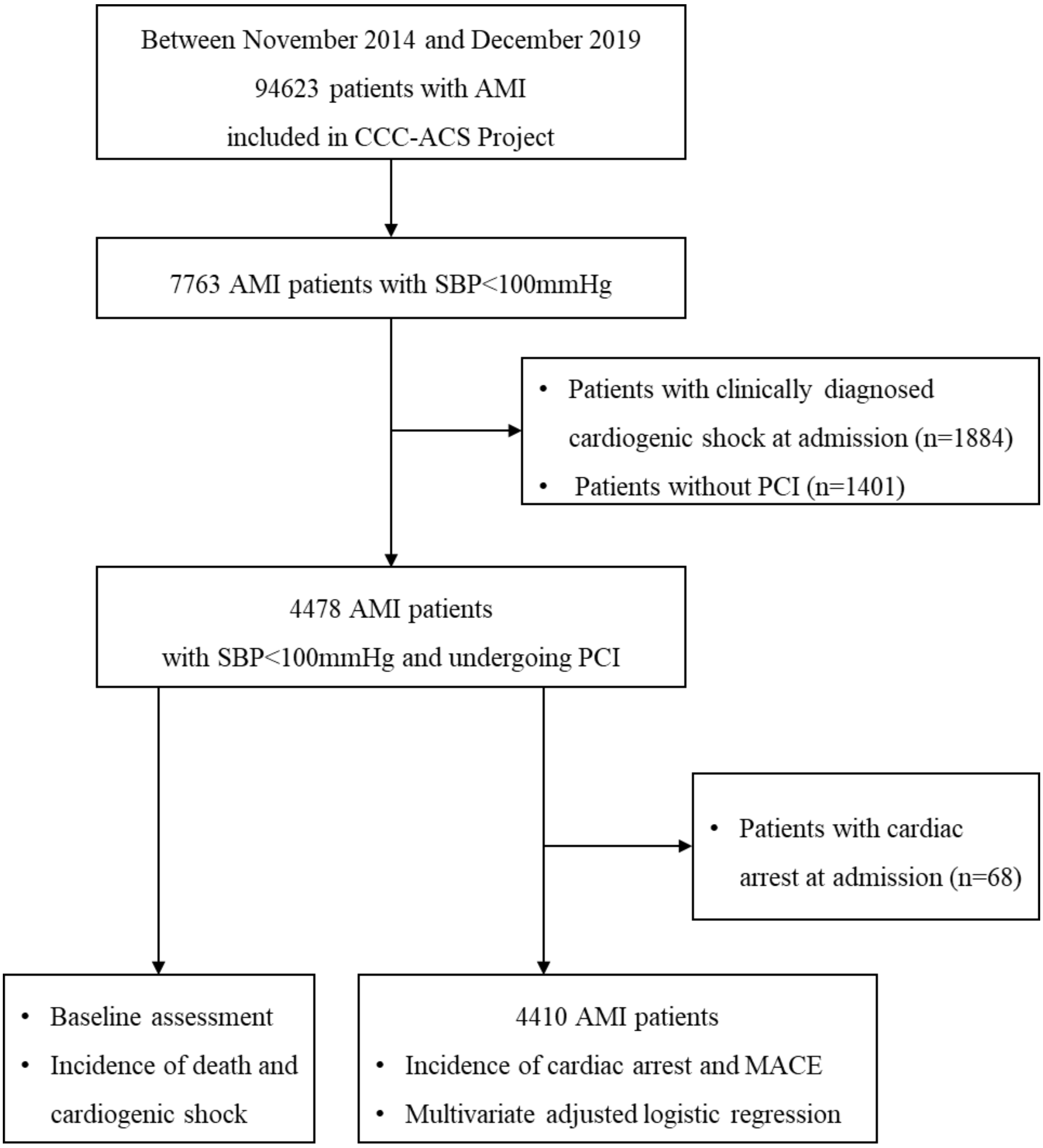

Supplementary Figure 1 Flow chart of study population

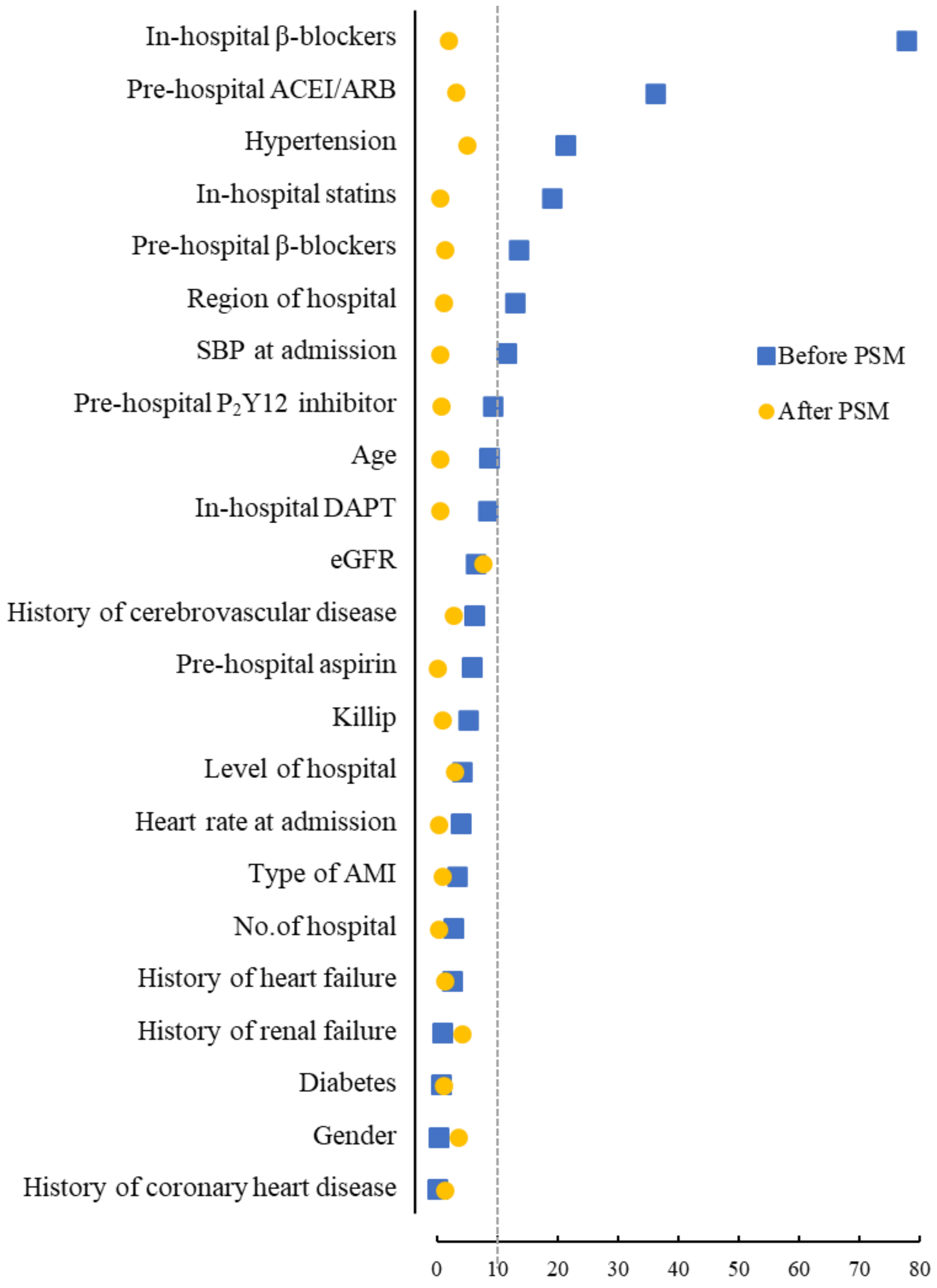

Supplementary Figure 2 Absolute Standardized Differences before and after Propensity Score Matching
